# Health Benefits of Probiotics in Sport and Exercise - Non-existent or a Matter of Heterogeneity? A Systematic Review

**DOI:** 10.3389/fnut.2022.804046

**Published:** 2022-02-23

**Authors:** Melina Heimer, Marc Teschler, Boris Schmitz, Frank C. Mooren

**Affiliations:** ^1^Department of Rehabilitation Sciences, Faculty of Health, University of Witten/Herdecke, Witten, Germany; ^2^DRV Clinic Königsfeld, Center for Medical Rehabilitation, Ennepetal, Germany

**Keywords:** exercise, gut microbiota, respiratory infection, immune system, nutrition, immunology, probiotic, gastrointestinal

## Abstract

**Background:**

The use of probiotics in sports has been growing in recent years, as up to 50% of athletes suffer from training- and performance-limiting gastrointestinal (GI) problems. Moreover, repeated exhaustive exercise and high training loads may lead to a transiently depressed immune function, associated with an increased risk of upper respiratory tract infection (URTI).

**Aim:**

To provide a qualitative analysis of probiotic effects on URTI, GI symptoms and the immune system in healthy individuals under consideration of performance level as main classifier.

**Methods:**

A systematic review of the literature was conducted (PubMed, SPORTDiscus with Full Text, Web of Science) to analyze the effects of probiotics in athletes and healthy active individuals on GI problems, URTI, and the immune system. A qualitative synthesis with performance level and treatment duration as main classifiers was performed.

**Results:**

Of 41 eligible studies, 24 evaluated the effects of probiotic supplements in athletes, 10 in recreationally active individuals and 7 in healthy untrained adults. Large heterogeneity was observed in terms of probiotic strains, mode of delivery, performance level, treatment duration and outcome assessment. Overall, studies provided inconsistent observations.

**Conclusion:**

The effects of probiotics on immune system, URTI, and GI symptoms in athletes, healthy adults and recreationally active individuals remain inconclusive. Based on the analyzed studies and identified parameters, this article provides suggestions to align future research on the effects of probiotics in exercise.

**Systematic Review Registration:**

PROSPERO, identifier: CRD42021245840.

## Introduction

The microbiota (i.e., the communities of commensal, symbiotic and pathogenic microorganisms) is mainly affected by dietary composition and has a significant impact on health (1). Also referred to as commensals, part of the gut microorganisms exert specific beneficial effects on the body including improved micro- and macronutrient uptake (2), increased intestinal barrier function, intestinal epithelial cell regeneration, modulation of the immune system, and improved mucosal barrier (3). Imbalance of the gut microbiota is associated with gastroenteric disorders, respiratory illness as well as metabolic and cardiovascular diseases (4). The Food and Agriculture Organization of the United Nations/World Health Organization (FAO/WHO) defines probiotics as “live microorganisms which when administered in adequate amounts confer a health benefit on the host” (5) with gut and immune health being the predominant applications (6–8). Notably, the term “probiotic” is frequently used for dietary supplements consisting of preparations that contain a multitude of different viable microorganisms. However, probiotic benefits are strain-specific and some strains are more likely to improve health outcomes than others (9). Microorganisms are effectors of the immune system since their characteristic components [nucleic acids, proteins, lipopolysaccharides (LPS) and metabolites] represent potential antigens and toxins. By contrast, microorganisms exerting probiotic potential target immune and body cells and regulate immune responses in a beneficial way (10). Lately, the specific microbial-derived metabolites, such as short-chain fatty acids (SCFAs), tryptophan, and retinoic acid, have been described to play a central regulatory role in the interaction of the host's immune response (11). Moreover, SCFAs may act to improve gut barrier function (12).

Gut microbiota compositions differ in athletes compared to sedentary individuals, as the human gut may be influenced by physical activity levels as well as training intensity and competition level (12). In addition, athlete's diet usually differs from the general population in terms of carbohydrate and protein intake, exerting additional effects on gut microbiota (10, 13). Especially endurance athletes may be exposed to extreme physiological conditions that put high stress on the body and affect normal organ function and homeostasis. Consequently, a high prevalence of upper respiratory tract infections (URTI) (14) and gastrointestinal (GI) problems (15) has been documented, the latter being associated with increased permeability of the gastrointestinal epithelial wall and disruption of mucous thickness and higher rates of bacterial translocation (4). Imbalance in gut microbiota may thus limit athlete's training performance, competitiveness, and overall wellbeing also including fatigue (16), depression and anxiety (17). Of note, 30–50% of athletes experience one or more GI symptoms during competitive events (13) including heartburn, indigestion, bloating or constipation. Serious medical conditions such as ischemic bowel, hemorrhagic gastritis, and hematochezia may also occur (14, 15). Reasons for GI symptoms may involve physiological, mechanical, or nutritional effectors, and reduced mesenteric blood flow, since the blood supply to the GI tract decreases by 60 to 70% already at exercise intensities of 70% of maximum oxygen consumption (VO_2max_) (14–17). By contrast, moderate exercise increases the number of health-promoting bacteria which produce SCFAs, inducing positive physiological effects (18, 19). Another common condition in athletes are URTIs, which account for 35–65% of disease-associated presentations in sports medicine clinics (20). URTI includes infections of the pharynx, sinuses, the middle ear, or the tonsils and is a mainly caused by various viruses such as respiratory viruses, rhinovirus, influenza, and corona virus interacting with the mucosa of the upper airways (21). Further reasons can be bacterial infections, allergic responses, undiagnosed asthma, and exercise-related trauma (20). The high rate of URTI in athletes has been linked to the observation that exhaustive exercise can lead to systemic immunosuppression (22) and oxidative stress (23), including reduced natural killer (NK) cells and T-lymphocytes (9). Even though acute exercise is known to increase the numbers of neutrophils and monocytes, the immune function can be reduced during recovery, as part of the physiological stress response to exercise (24), a phenomenon known as the “open window” in which athletes may be prone to infections. Further, the anti-inflammatory cytokines interleukin (IL)-10, IL-1Ra, soluble tumor necrosis factor receptor (sTNFR) and inflammation-responsive cytokine IL-6 increase by exhaustive exercise (9). More recently, changes in the gut microbial composition have been linked to local alterations in immune response and development in the respiratory tract (25) in that the lungs production of type-I interferons (IFNs), which prevent virus infections, may be increased (26, 27).

As probiotics have been reported to affect the immune system by inhibition of NK cell activity (28) induction of anti-inflammatory IL-10 (29) and IFN-γ secretion as well as salivary cortisol and gut immunoglobulin (IgA) levels (30) in addition to T and B cell activation (28), they may induce beneficial effects on URTI incidence and severity. In addition, probiotic-induced fermentation of fibers increases the production of SCFAs and may thus strengthen the gut barrier function with beneficial effects on GI problems during endurance exercise. With regard to the potentially improved resistance to illness and immunomodulative effects of probiotics, several studies have investigated probiotic effects in athletes. However, the effectiveness of probiotics in physical exercise on the immune system, URTI and GI problems is still a matter of debate.

### Objective

Based on the hypothesis that probiotics may exert differential effects in athletes compared to recreationally active individuals, the aim of this systematic review was to provide a structed summary using a qualitative analysis of studies investigating the effects of probiotics on URTI, GI symptoms and the immune system in healthy individuals under consideration of performance level as main classifier. We propose that this approach would be effective to reveal the specific beneficial potential of probiotics in sport and exercise.

## Methods

### Study Design and Participants Eligibility Criteria

We performed a systematic review (PROSPERO, CRD42021245840) in accordance with the PRISMA guidelines and following the suggestions for reporting on qualitative summaries (31, 32). Any original article reporting on probiotics in physical exercise was considered for the analysis. Included studies had to report on URTI, GI (as defined by authors; symptoms/severity and duration) or markers of the immune system including IL-6, IL-8, IL-10, granulocyte macrophage-colony stimulating factor (GM-CSF), IFN-γ, TNF-α, and IL-1Ra, serum concentrations of tryptophan, phenylalanine, kynurenine, tyrosine, neopterin, C-reactive protein (CRP), protein carbonyl content (PCC), malondialdehyde (MDA) and total oxidation status of lipids (TOS). Only articles available as full-text (after an attempt to contact the corresponding author) reporting on healthy humans aged 14–65 years were included. This was done since different effects of probiotics and physical activity on the included outcome variables may be observed in children. Articles were not eligible if they (1) reported on probiotic use in patients or animals, (2) did not investigate effects of probiotics only/ included additional interventions, and (3) were not original research [a review or book (chapter)]. Articles were excluded if they (1) focused on performance only, (2) were not written in English (full text), (3) were gray literature or website articles, and (4) did not clearly report on included subjects, interventions, outcome measures and statistical analysis. The combined used of probiotics, prebiotics and synbiotics was not an exclusion criterium. The eligibility criteria were selected also in accordance with the quality assessment (see below).

### Search Strategy and Data Sources

**Figure 1:**
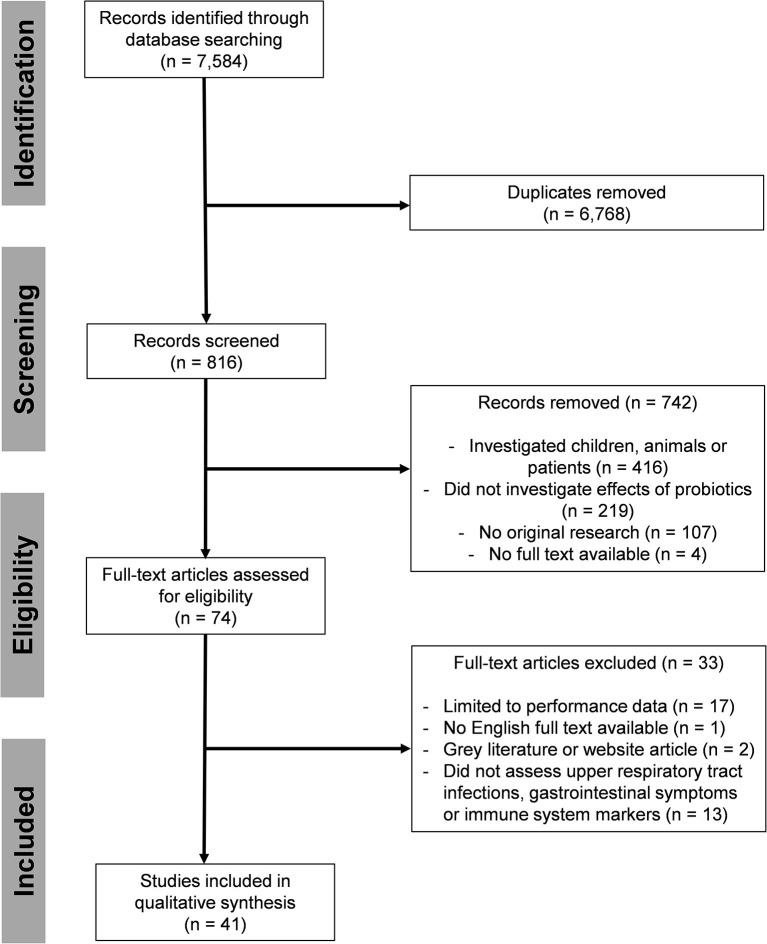
PRISMA flow chart.

Electronic searches were performed based on the PICO criteria. Studies in athletes, recreationally active individuals and healthy adults in risk of URTI, GI and transiently depressed immune function [P] in which probiotic foods or supplements [I] were compared to controls not receiving probiotics [C] for establishment of significant effects on URTI, GI or markers of the immune system [O] were included. A systematic search of the literature was conducted (MH) using PubMed, SPORTDiscus with Full Text, and Web of Science for records published until March 2021. Databases were searched using variations and combination of the following keywords: “probiotic,” “exercise,” “gut microbiota,” “respiratory tract infection,” “immune system,” “sport,” “immunology,” “athlete,” “physical activity,” “recreational,” “gastrointestinal disease,” “URTI,” “inflammation.” Additional filters were used if applicable. The search syntax used for the individual data bases is presented in Supplementary Table 1. Manual searches were also performed using reference lists from identified articles and available reviews. The individual steps of report identification, screening and processing are documented in the PRISMA flow chart (Figure 1) (33). Search results and fulfillment of eligibility criteria were discussed if unclear (MH and BS) until consensus was achieved and upon disagreement, a third person was consulted to determine inclusion.

### Study Selection and Data Extraction

Data were extracted by two reviewers (MH and BS) and tables were created including information on first author, year of publication, probiotic type, strain, dose, and mode of delivery, participants (total number, sex), study type, main outcome, influence on URTI, GI and immune system defined as significant increase, decrease or insignificant effect, type of data assessment, type of sport/ exercise, contextual/ setting factors (acute/ chronic testing), and additional observation.

### Grouping of Studies and Synthesis

To provide a structured qualitative summary, studies were grouped by main categories treatment duration and activity/ performance level including (1) professional/ amateur athletes, (2) recreationally active adults, and (3) previously sedentary (healthy) adults. Heterogeneity was investigated using ordering tables including URTI, GI or immune system as main outcome and the above-mentioned categories. Certainty of the evidence was addressed using an evaluation of how directly the included studies addressed the planned question/ applied methodology (measurement validity), the number of studies and participants and the consistency of effects across studies.

### Definitions

Individuals were classified as athletes (professional level and non-professional amateurs), recreationally active individuals and healthy adults (previously sedentary), based on the author's descriptions. URTI and GI symptom definitions were accepted as defined by authors. In case of imprecise, uncommon, unclear/ conflicting, or missing descriptions of test participants, full texts were screened by two reviewers (MH and BS) for additional information including club or union associations, training patterns or other information.

### Quality Assessment

The methodological quality of the studies was assessed using the 11-item PEDro scale based on the Delphi list developed by Verhagen and colleagues (34). The individual items can be scored as “yes” or “no.” For our analysis, we determined (in accordance with the above mentioned inclusion criteria) that the following items (6 out of 11) had to be scored “yes”: eligibility criteria were specified; the groups were similar at baseline regarding the most important prognostic indicators (in case of non-randomized controlled trial (RCT) study design this item was not applicable); measures of at least one key outcome were obtained from more than 85% of the subjects initially allocated to groups/intervention; all subjects for whom outcome measures were available received the treatment (or control condition) as allocated or, where this was not the case, data for at least one key outcome was analyzed by “intention to treat;” the results of (between-group) statistical comparisons were reported for at least one key outcome; the study provided both point measures and measures of variability for at least one key outcome. Studies were rated by two reviewers (MH and BS). Disagreements were resolved by discussion if necessary. The researchers were not blinded to study authors, results, or publication journal.

## Results

A total of 41 articles met the eligibility criteria, involving 2,189 participants. Twenty-four studies evaluated the effects of probiotic supplements in athletes, 10 studies in recreationally active individuals and 7 studies in healthy untrained adults. Included studies had an average score of 9/10 on the PEDro scale indicating low to moderate risk of bias (Supplementary Table 2). Thirty-one studies were conducted as RCT, 7 had a crossover design and three were longitudinal studies with pre-post analysis. Sixteen studies investigated the effects on URTI (8 found significant positive effects), 16 on GI symptoms (5 found significant positive effects) and 31 on the immune system (14 found significant positive effects). An analysis of the intervention period revealed a mean intervention time of ~8 weeks and a maximal intervention time of 21 weeks. Thus, studies were categorized by intervention time <5 weeks (18 studies), 5–12 weeks (14 studies) and >12 weeks (9 studies).

**Figure 2:**
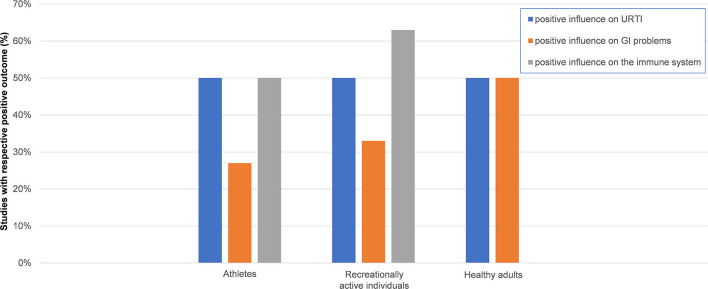
Effect of probiotic supplementation on URTI, GI problems and the immune system by performance level. Columns illustrate the ratio of studies reporting positive effects on upper respiratory tract infections (URTI), gastrointestinal (GI) problems and the immune system. Results were based on the following number of studies. URTI in athletes, 5/10 studies; GI problems in athletes, 3/11 studies; immune system in athletes, 9/18 studies; URTI in recreationally active individuals, 2/4 studies; GI problems in recreationally active individuals, 1/3 studies; immune system in recreationally active individuals, 5/8 studies; URTI in healthy adults, 1/2 studies; GI problems in healthy adults, 1/2 studies; immune system in healthy adults, 0/5 studies. No influence was defined as non-significant change in outcome variables of URTI, GI problems or the immune system. GI problems were defined as incidence of symptoms and severity of nausea, vomiting, diarrhea, abdominal pain, abdominal bloating, flatulence, stomach “rumbles,” loss of appetite, and markers for GI barrier function (e.g., zonulin); URTI was defined as symptoms and severity of throat soreness, sneezing, fever, ear pain, blocked or runny nose, cough, duration, number of episodes based on author's descriptions.

In athletes, 50% of the studies found significant positive effects on URTI, 27% on GI problems, and 50% on the immune system (Figure 2). In recreationally active individuals, 50% of the studies reported significant positive effects on URTI, 33% on GI problems, and 63% on the immune system. In healthy adults, 50% of the studies detected significant positive effects on URTI and 50% on GI problems, while no study out of five in healthy adults reported significant positive effects on the immune system (Figure 2).

**Figure 3:**
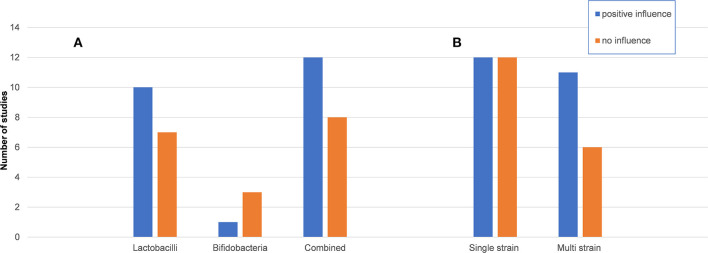
Effect of probiotic supplementation on URTI, GI problems and the immune system by probiotic strain. Columns illustrate the number of studies reporting positive effects on upper respiratory tract infections (URTI), gastrointestinal (GI) problems or the immune system for the different probiotic **(A)** genera and their combination and **(B)** single or multi strain preparations. *Lactobacilli*, preparations only including *Lactobacillus* strains; *Bifidobacteria*, preparations only including *Bifidobacterium* strains; combined, including different *Lactobacillus* and *Bifidobacteria* strains with or without different other genera; single strain, only one strain of *Lactobacillus* or *Bifidobacteria* was given; multi strains, any combination of different *Lactobacillus* and/ or *Bifidobacteria* was given. Positive influence was defined as any significant positive effect on URTI, GI and the immune system based on author's descriptions.

In terms of probiotics applied, different combinations of *Lactobacilli* and/ or *Bifidobacteria* in form of single or multi strains, sometimes combined with *S. thermophilus* or *E. faecium* were used (Table 1). Seventeen studies used *Lactobacillus* strains (10 detected positive effects), 4 studies used *Bifidobacterium* (one found positive effects), and 20 studies used a combination with or without excipients (12 reported significant positive effects) (Figure 3). Twenty-four studies used single strain probiotics (12 detected positive effects) and 17 used multi strain probiotics (11 reported positive effects). In addition, different modes of delivery were applied. Sixteen studies provided (enterically-coated) capsules and 25 used a different mode of delivery without protection from dissolution or disintegration in the gastric environment (mainly solutions in water/ milk or yogurt). Of these, 9 (56%) and 12 (48%) reported positive effects, respectively (Table 2).

**Table 1:** Type, dose, and duration of probiotic use of included studies.

**References**	**Type (strain and serving), dose and duration**
**Athletes**	
Salehzadeh (35)	200 ml of probiotic yogurt drink containing *S. thermophilus or L. delbrueckii ssp. Bulgaricus*, 1 × 10^5^ CFU daily; 4 weeks
Brennan et al. (36)	*L. salivarius*, dose not indicated, daily; 4 weeks
Tiollier et al. (37)	*L. casei*, dose not indicated; 3 weeks
Huang et al. (29)	*L. plantarum*, 3 × 10^10^ CFU daily, 3–4 weeks
Gill et al. (38)	*L. casei*, 10 × 10^10^ CFU daily; 1 week
Gill et al. (39)	*L. casei*, 10 × 10^10^ CFU daily; 1 week
Haywood et al. (40)	*L. gasseri*, 2.6 × 10^9^ CFU, *B. bifidum and B. longum*, 0.2 × 10^9^ CFU daily; 4 weeks
Sashihara et al. (41)	*L. gasseri*, 1 × 10^9^ CFU or alpha-lactalbumin, 900 mg, and *L. gasseri*, 1 × 10^9^ CFU, 3x daily; 4 weeks
Shing et al. (42)	*L. acidophilus, L. rhamnosus, L. casei, L. plantarum, L. fermentum, B. lactis, B. breve, B. bifidum, S. thermophilus*, 4.5 × 10^10^ CFU daily; 4 weeks
West et al. (43)	*B. animalis ssp. lactis*, 2 × 10^10^ CFU or *L. acidophilus* and *B. animalis ssp. lactis*, 5 × 10^9^ CFU daily; 5 months
Townsend et al. (44)	*B. bifidum, B. lactis, E. faecium, L. acidophilus, L. brevis*, and *L. lactis*, 2.5 × 10^9^ CFU; 12 weeks
Gepner et al. (45)	Combined supplementation of *B. coagulans*, 1.0 × 10^9^ CFU and HMB, 3 g daily; 6 weeks
Carbuhn et al. (46)	*B. longum*, 1 × 10^9^ CFU daily; 6 weeks
Charlesson et al. (47)	*L. acidophilus, B. lactis, L. rhamnosus*, dose not indicated, daily; 8 weeks
Strasser et al. (48)	*B. bifidum, B. lactis, E. faecium, L. acidophilus, L. brevis, and L. lactis*, 2.5 × 10^9^ CFU; 12 weeks
Marshall et al. (49)	Different combinations of *L. acidophilus*, 10 × 10^9^ CFU, *L. acidophillus*, 10 × 10^9^ CFU, *B. bifidum*, 9.5 × 10^9^ CFU, *B. animalis ssp. lactis*, 0.5 × 10^9^ CFU, and 55.8 mg fructooligosaccharides or *L. acidophilus*, 2 × 10^9^ CFU, *L. acidophilus*, 2 × 10^9^, *B. bifidum*, 0.5 × 10^9^ CFU, *B. animalis ssp. lactis*, 0.95 × 10^9^ CFU, *L. salivarius*, 5 × 10^9^ CFU; each 5-g dose also contained 0.9 g L-glutamine; 12 weeks
Salarkia et al. (50)	Probiotic yogurt containing *L. acidophilus, L. delbrueckii bulgaricus, B. bifidum*, and *S. salivarus thermnophilus*, 4 × 10^10^ CFU daily; 8 weeks
Cox et al. (51)	*L. fermentum*, 1.2 × 10^10^ CFU daily; 8 weeks
Michalickova et al. (52)	*L. helveticus lafti*, 2 × 10^10^ CFU daily; 14 weeks
Michalickova et al. (53)	*L. helveticus lafti*, 2 × 10^10^ CFU daily; 14 weeks
O'Brien et al. (54)	Kefir, probiotic strain and amount not indicated, 2x/day; 15 weeks
Gleeson et al. (55)	*L. casei shirota*, 6.5 × 10^9^ CFU; 20 weeks
Lamprecht et al. (56)	*B. bifidum, B. lactis, E. faecium, L. acidophilus, L. brevis*, and *L. lactis*, 1 × 10^10^ CFU daily; 14 weeks
Pumpa et al. (57)	Ultrabiotic 60, SB Floractiv 250 mg, 2x/day; 17 weeks
**Recreational exercise**	
Jäger et al. (9)	*B. breve*, 5 × 10^9^ AFU, *S. thermophilus*, 5 × 10^9^ AFU daily; 2 weeks
Vaisberg et al. (58)	Fermented milk beverage containing *L. casei shirota*, 4 × 10^10^ CFU daily; 4 weeks
Komano et al. (59)	Heat-inactivated dried powder of *L. lactis*, 1 capsule per day, 2 weeks
Pugh et al. (60)	*L. acidophilus, B. bifidum, B. animalis subs p. lactis*, > 25 billion CFU daily in total; 4 weeks
Martarelli et al. (23)	*L. rhamnosus* and *L. paracasei*, 1 × 10^9^ CFU daily; 4 weeks
Moreira et al. (61)	*L. rhamnosus*, milk-based drink, 4 × 10^10^ CFU daily; 12 weeks
Kekkonen et al. (62) same subject as Moreira et al.	*L. rhamnosus*, milk-based drink, 4 × 10^10^ CFU daily; 12 weeks
Välimäki et al. (11)	*L. rhamnosus*, 4 × 10^10^ CFU daily; 12 weeks
Roberts et al. (10)	*L. acidophilus*, 10 × 10^9^ CFU, *L. acidophillus*, 10 × 10^9^ CFU, *B. bifidum*, 9.5 × 10^9^ CFU, *B. animalis ssp. lactis*, 0.5 × 10^9^ CFU and 55.8 mg fructooligosaccharides and 400 mg alpha-lipoic acid and 600 mg N-acetylcarnitine daily; 12 weeks
Gleeson et al. (63)	*L. casei shirota*, 6.5 × 10^9^ CFU, 2x daily; 16 weeks
**Healthy adults**	
Hoffmann et al. (12)	Inactivated *B. coagulans*, dose not indicated; 2 weeks
Meng et al. (64)	One 240 g serving of yogurt smoothie per day, added log 10 ± 0.5 CFUs/day or *B. animalis ssp. lactis* capsule; 4 weeks
Mooren et al. (65)	5 ml of an *E. coli Nissle 1917* suspension daily; 4 weeks
Muhamad and Gleeson (66)	*L. acidophilus, L. delbrueckii ssp. bulgaricus, L. lactis ssp. lactis, L. casei, L. helveticus, L. plantarum, L. rhamnosus, L. salivarius ssp. salivarius, B. breve, B. bifidum, B. infantis, B. longum, B. subtilis*, and *S. thermophilus*, 6 × 10^9^ CFU daily; 4 weeks
Ibrahim et al. (67)	*L. acidophilus, L. lactis, L. casei, B. longum, B. bifidum* and *B. infantis* twice daily; 3 × 10^10^ CFU; 12 weeks
West et al. (68)	*L. paracasei ssp. paracasei*, 4.6 × 10^8^ CFU, *B. animalis ssp. lactis*, 6 × 10^8^ CFU, *L. acidophilus*, 4.6 × 10^8^ CFU, *L. rhamnosus*, 4.6 × 10^8^ CFU, daily; 3 weeks
West et al. (69)	*B. animalis ssp. lactis*, 2 × 10^10^ CFU, or *L. acidophilus* and *B. animalis ssp. lactis*, 7.5 × 10^9^ CFU daily; 5 months

*AFU, Active Fluorescent Unit; CFU, colony forming units; HMB, Beta-hydroxymethylbutyrate; SB, Saccharomyces boulardii; ssp, subspecies*.

**Table 2:** Effects of probiotics by performance level and duration of intervention.

**Athletes**								
**Reference**	**Strain (Mode of delivery)**	**Sample (** ***n*** **)**	**Study type (** ***n*** **)**	**Sex**	**Main outcome**	**Influence on**			**Additional observation**
						**URTI**	**GI**	**Immune system**	
**Intervention** **<** **5 weeks**									
Salehzadeh (35)	Single (probiotic yogurt)	30	RCT (PRO = 15, CTRL = 15)	male	Decrease in resting CRP levels, increase in resting HDL levels, no effects on cholesterol, triglyceride and LDL levels	n.a.	n.a.	↑	Increased VO_2max_
Brennan et al. (36)	Single (n.a.)	7	RCT (PRO = 3, CTRL = 4)	male	Reduced resting urine sucrose levels, increase in microbial diversity	n.a.	↑	n.a.	-
Tiollier et al. (37)	Single (fermented milk)	47	RCT (PRO = 24, CTRL = 23)	male	No effect on resting IgA levels, no effect on respiratory infection incidence/ symptoms duration^#^	↔	n.a.	↔	-
Huang et al. (29)	Single (capsule)	34	RCT (PRO = 17, CTRL = 17)	male	Reduced resting TNF-alpha, IL-6, IL-8 levels, reduced oxidative stress (creatine kinase, thioredoxin, and myeloperoxidase indices), increased resting IL-10 levels, increased amino acids	n.a.	n.a.	↑	Increased peak anaerobic power, mean power, VO_2_max and decreased fatigue index
Gill et al. (38)	Single (probiotic beverage)	8	Crossover	male	No change in resting circulatory endotoxin concentration or plasma cytokine profile	n.a.	n.a.	↔	-
Gill et al. (39)	Single (probiotic beverage)	8	Crossover	male	No change in resting S-lysozyme response and S-antimicrobial protein, no effect on resting oral/respiratory mucosal immune protection	↔	n.a.	↔	-
Haywood et al. (40)	Multi (n.a.)	30	Crossover	male	Reduced resting GI and URTI episodes*, no effect on URTI and GI symptom severity	↑	↑	n.a.	-
Sashihara et al. (41)	Multi (tablet)	44	RCT (PRO = 14, CTRL = 30)	male	Reduced acute NK cell activity post-exercise	n.a.	n.a.	↑	Elevated mood from depression state, minor resting fatigue
Shing et al. (42)	Multi (capsule)	10	Crossover	male	No effect on serum LPS, IL-6, IL-10, IL-1ra, levels; no effect on gastrointestinal permeability and GI symptoms^#^	n.a.	↔	↔	Increased running time to fatigue
**Intervention 5–12 weeks**									
West et al. (43)	Single (capsule)	97	RCT (PRO = 47, CTRL = 50)	male = 62, female = 35	No effect on URTI or GI symptoms*	↔	↔	n.a.	Decreased duration and severity of respiratory illness in men, increased in women
Townsend et al. (44)	Single (capsule)	25	RCT (PRO = 13, CTRL = 12)	male	Decreased resting TNF-α levels, no effect on zonulin	n.a.	↔	↑	-
Gepner et al. (45)	Single (capsule)	26	RCT (PRO = 9, CTRL = 17)	male	Decreased resting IL-6 and IL-10 levels	n.a.	n.a.	↑ ↓	Maintained muscle integrity
Carbuhn et al. (46)	Single (capsule)	17	RCT (PRO = 8, CTRL = 9)	female	No effect on IFN-γ, IL-2, IL-4, IL-6, IL-10, TNF-α and salivary immunoglobulin A levels, no effect on gut inflammation markers (endotoxin/LPS, LPS binding protein)	n.a.	↔	↔	Significantly improved recovery^§^
Charlesson et al. (47)	Multi (n.a.)	8	Longitudinal	male	No effect on eubacteria, bifidobacteria, bacteroides, and SCFA concentrations	n.a.	↔	n.a.	-
Strasser et al. (48)	Multi (powder)	33	RCT (PRO = 17, CTRL = 16)	male = 13, female = 16	reduced URTI symptoms, no reduction of GI symptoms^#^	↑	↔	n.a.	Lowered post-exercise tryptophan levels in placebo but not probiotic group
Marshall et al. (49)	Multi (capsules)	32	RCT (PRO = 21, CTRL = 11)	male	No effect on extracellular Hsp72	n.a.	n.a.	↔	-
Salarkia et al. (50)	Multi (probiotic yogurt)	46	RCT (PRO = 23, CTRL = 23)	female	Reduction in respiratory and ear infections, no effect on GI episodes*	↑	↔	n.a.	-
**Intervention** **>** **12 weeks**									
Cox et al. (51)	Single (capsules)	20	Crossover	male	Reduction in respiratory symptoms and severity*, increased resting INF-γ levels, no effect on salivary IgA levels and IL-4, IL-12 serum levels	↑	n.a.	↔	-
Michalickova et al. (52)	Single (capsules)	30	RCT (PRO = 15, CTRL = 15)	male = 24, female = 6	No difference on serum IgA, IgG, and IgM levels	n.a.	n.a.	↔	Elevated IgM levels in both groups
Michalickova et al. (53)	Single (capsule)	39	RCT (PRO = 20, CTRL = 19)	male	Reduction of URTI duration and symptoms*, improved resting CD4+/CD8+ ratio	↑	n.a.	↑	Increased self-rated sense of vigor
O'Brien et al. (54)	Single (kefir)	65	RCT (PRO = 34, CTRL = 31)	male and female (ratio undefined)	Attenuated increase in resting serum CRP levels	n.a.	n.a.	↑	Improved 1.5-mile time trial
Gleeson et al. (55)	Single (fermented milk)	243	RCT (PRO = 126, CTRL = 117)	male = 156, female = 112	Reduced cytomegalovirus and epstein baar virus load, no reduction in URTI episodes, duration, and severity^#^	↔	n.a.	↑	-
Lamprecht et al. (56)	Multi (powder)	23	RCT (PRO = 11, CTRL = 12)	male	Reduction in resting serum zonulin levels, no effect on CP levels	n.a.	↑	↔	-
Pumpa et al. (57)	Multi (capsule)	19	RCT (PRO = 9, CTRL = 10)	male	No effect on URTI and GI incidence and severity^#^, increase in resting salivary alpha-amylase	↔	↔	↑	-
**Recreationally active individuals**									
**Intervention** **<** **5 weeks**									
Jäger et al. (9)	Single (probiotic beverage)	15	Crossover	male	No effect on post-exercise IL-6 levels	n.a.	n.a.	↔	-
Vaisberg et al. (58)	Single (fermented milk)	42	RCT (PRO = 20, CTRL = 22)	male	No differences in duration of URTI^#^; increased resting IL-10 levels, decreased resting IL-1, IL-5, IL-6, IL-13, and TNF- α levels	↔	n.a.	↑	-
Komano et al. (59)	Multi (capsule)	51	RCT (PRO = 26, CTRL = 25)	male	Lower days and symptoms of URTI ^#, $^, increased resting CD86 levels	↑	n.a.	↑	Fewer days of fatigue
Pugh et al. (60)	Multi (capsule)	20	RCT (PRO = 11, CTRL = 9)	male and female (ratio undefined)	Reduced resting GI symptoms and severity‡	n.a.	↑	n.a.	-
Martarelli et al. (23)	Multi (powder)	24	RCT (PRO = 12, CTRL = 12)	male	Increased resting plasma antioxidant levels	n.a.	n.a.	↑	-
**Intervention 5–12 weeks**									
Moreira et al. (61)	Single (milk-based drink)	141	RCT (PRO = 70, CTRL = 71)	male = 123, female = 16	No effects on atopy or asthma symptoms	n.a.	n.a.	↔	-
Kekkonen et al. (62)	Single (milk-based drink)	141	RCT (PRO = 70, CTRL = 71)	male = 123, female = 16	No effect on number of respiratory infections or GI-symptom episodes^#^	↔	↔	n.a.	-
Välimäki et al. (11)	Single (milk-based drink)	119	RCT (PRO = 61, CTRL = 58)	male = 105, female = 14	No effect on ox-LDL, s-TRAP, or serum antioxidant levels	n.a.	n.a.	↔	-
Roberts et al. (10)	Multi (capsule)	30	RCT (PRO = 20, CTRL = 10)	male = 25, female = 5	No effect on GI permeability, GI severity or symptoms^#^, reduced resting endotoxin level	n.a.	↔	↑	-
**Intervention** **>** **12 weeks**									
Gleeson et al. (63)	Single (fermented milk)	54	RCT (PRO = 42, CTRL = 42)	male = 54, female = 30	Lower URTI episodes^#^, higher resting saliva IgA concentration	↑	n.a.	↑	-
**Healthy adults**									
**Intervention** **<** **5 weeks**									
Hoffmann et al. (12)	Single (powder)	16	RCT (PRO = 8, CTRL = 8)	male	No effect on IL-6, IL-10, IFN-γ and TNF-α levels	n.a.	n.a.	↔	-
Meng et al. (64)	Single (probiotic yogurt)	30	Crossover	male = 11, female = 19	No reduction in URTI severity^#^, no effect on IL-2 levels and NK-cell cytotoxicity	↔	n.a.	↔	Elevated IL-2 levels and NK-cell cytotoxicity in all groups
Mooren et al. (65)	Single (probiotic beverage)	19	Test/re-test	male	Reduced increase in acute I-FABP and TBARS after exercise, no effects on zonulin, CLDN3, and LPS	n.a.	↑	n.a.	-
Muhamad and Gleeson (66)	Multi (capsule)	11	Longitudinal	male	No effect on S-IgA, alpha-amylase, lactoferrin, and lysozyme concentrations	n.a.	n.a.	↔	-
**Intervention 5–12 weeks**									
Ibrahim et al. (67)	Multi (powder)	48	RCT (PRO = 24, CTRL = 24)	male	No effect on IL-10 levels	n.a.	n.a.	↔	IL-10 concentration increased in both groups
West et al. (68)	Multi (capsules)	22	RCT (SYN = 11, PRE = 11)	male	No effect on GI permeability, no effect on SCFA, salivary lactoferrin and serum cytokines	n.a.	↔	↔	-
**Intervention** **>** **12 weeks**									
West et al. (69)	Multi (powder)	465	RCT (PRO = 316, CTRL = 149)	male = 241, female = 224	Reduced URTI episodes^#^	↑	n.a.	n.a.	Insufficient GI illness episodes for analysis

Gastrointestinal (GI) problems were defined as incidence of symptoms and severity of nausea, vomiting, diarrhea, abdominal pain, abdominal bloating, flatulence, stomach “rumbles,” loss of appetite, as well as markers for GI barrier function (e.g., zonulin); Upper respiratory tract infection (URTI) was defined as symptoms and severity of throat soreness, sneezing, fever, ear pain, blocked or runny nose, cough, duration, number of episodes; ↔, no effect on outcome; ↑, significant positive effect on outcome; ↓, significant negative effect on outcome; #, by questionnaire; ^*^, by diary; $, by self-reported symptom score; §, by Recovery-Stress Questionnaire for Athletes; ‡, by Gastrointestinal Symptom Rating Scale (70); CD, cluster of differentiation; CLDN3, claudin 3; CRP, human C-reactive protein; CTRL, control group; GI, gastrointestinal; HDL, high-density lipoprotein; Hsp72, heat shock protein 72; I-FABP, intestinal fatty acid-binding protein; IFN-γ, interferon-gamma; Ig, immunoglobulin; IL, interleukin; LDL, low-density lipoprotein; LPS, lipopolysaccharide; n.a., not assessed; NK cells, natural killer cells; ox-LDL, oxidized low-density-protein; PRE, prebiotic group; PRO, probiotic group; RCT, randomized controlled trial; S, secretory; SCFA, short-chain fatty acid; S-TRAP, serum antioxidant potential; SYN, synbiotic group; TBARS, thiobarbituric acid reactive substances; TNF-alpha, tumor necrosis factor alpha; URTI, upper respiratory tract infections; VO_2max_, maximal oxygen consumption.

### Probiotic Supplementation in Athletes

Sixty percent of the identified studies (24 of 41 studies) investigated the impact of probiotics on URTI (10 studies), GI symptoms (10 studies), and/or immune system (18 studies) in athletes. Outcomes were evaluated after supplementation for <5 weeks (*N* = 9), 5–12 weeks (*N* = 8) and > 12 weeks (*N* = 7). Five studies found significant positive effects of probiotics on URTI, 3 on GI symptoms and 10 on the immune system and inflammatory response.

#### Effects on URTI

As URTI are common in athletes, 10 studies investigated the effect of probiotic supplementation on URTI with 5 studies reporting significant positive effects (Table 2, Figure 2). All studies documented URTI via self-reported symptoms using questionnaires or daily diaries, recording the number, duration and severity of symptoms including throat soreness, sneezing, fever, ear pain, a blocked or runny nose and cough. One study defined two or more symptoms on at least two consecutive days as an episode of illness (43). No study involved medical diagnosis of URTI. Mucosal immunity was assessed by lactoferrin, lysozyme, secretory immunoglobulin (sIgA) and albumin concentrations.

Studies reporting reduced number, duration, and severity of infections included rugby players (*n* = 30, multi strain probiotic, 4 weeks) (40) and athletes in general (13 males and 16 females, no type of sport indicated, multi strain probiotics, 12 weeks) (48). A reduction in respiratory and ear infections has been documented in female endurance swimmers (*n* = 46, multi strain probiotic, 8 weeks) (50). Elite distance runners (*n* = 20, single strain probiotic, 16 weeks) showed a reduction in respiratory episodes and severity (51). A reduction in URTI duration and symptoms but not in severity and incidence was recorded in elite athletes involved in different sports (*n* = 39, single strain probiotic, 14 weeks) (53).

No effects were observed in endurance runners (*n* = 8, single strain probiotic, 7 days) (38), in commando cadets (*n* = 47, single strain probiotic, 3 weeks) (37), in university athletes and game players (*n* = 243, single strain probiotic, 20 weeks) (55) and elite rugby union athletes (*n* = 19, multi strain probiotic, 17 weeks) (57). One study reported sex-dependent differences in that no reduction of URTI has been found in female (*n* = 35) compared to male (*n* = 64) competitive cyclists (single strain probiotic, 11 weeks) (43).

#### Effects on GI Symptoms

Ten studies investigated the effects of probiotic supplementation on GI symptoms in athletes, 3 of which found significant positive effects. GI illness was measured by self-reported symptoms including nausea, vomiting, diarrhea, abdominal pain, abdominal bloating, flatulence, stomach “rumbles” and loss of appetite using scores for severity. Only one study (60) used the structured Gastrointestinal Symptom Rating Scale (70). Some studies involved fecal microbiology to determine abundance of *Bacteroides*, short chain fatty acids (SCFA) concentrations, α1-antitrypsin and zonulin levels (marker of GI barrier integrity/ intestinal permeability). One study (36) assessed GI permeability as sugar recovered in urine. Another study (47) used a daily log to record symptoms of diarrhea, classified using the World Health Organization (WHO) definition of ≥ 3 loose or watery stools in a 24-h period.

Significant changes in 60 bacterial species, an increased microbial diversity and an attenuated exercise-induced intestinal hyperpermeability indicated by urine sucrose concentration were found in endurance athletes (*n* = 7, single strain probiotics, 4 weeks) (36). Reduced episodes of GI illness were reported in rugby players (*n* = 30, multi strain probiotic, 4 weeks) (40). In a study involving 23 endurance-trained men (triathletes, runners, and cyclists; multi strain probiotic, 14 weeks) serum zonulin levels were decreased (56).

No effects on gut inflammation markers were found in division one collegiate female swimmers (*n* = 17, single strain probiotic, 6 weeks) (46). No effect on GI was observed in athletes (*n* = 33, multi strain probiotic, 12 weeks) (48), runners in the heat (*n* = 10, multi strain probiotic, 4 weeks) (42), division one baseball players (*n* = 25, single strain probiotic, 12 weeks) (44), athletes (no type of sport specified, *n* = 8, multi strain probiotic, 8 weeks) (47), elite rugby union athletes (*n* = 19, multi strain probiotic, 17 weeks) (57) and female endurance swimmers (*n* = 46, multi strain probiotic, 8 weeks) (50). Interestingly, number and duration of GI symptoms increased in 64 male competitive cyclists supplemented with probiotics (single strain, 11 weeks), which was interpreted as a response of the GI tract to alteration in the composition of microflora (43).

#### Effects on the Immune System

Eighteen studies investigated effects on the immune system and inflammatory response, 10 of which found significant positive effects. Frequently assessed markers included anti- and pro-inflammatory cytokines (IL-6, IL-8, IL-10, GM-CSF, IFN-γ, TNF-α, and IL-1RA), serum concentrations of tryptophan, phenylalanine, kynurenine, tyrosine, neopterin (immune activation marker), CRP and PCC, as well as MDA and TOS.

Positive effects were reported in endurance athletes (*n* = 30, single strain probiotic, 4 weeks) in terms of reduced CRP and increased high-density lipoprotein levels (35). In triathletes (*n* = 34, single strain probiotic, 3–4 weeks) reduced post-race inflammatory cytokine levels (TNF-α, IL-6, IL-8) and oxidative stress (thioredoxin and myeloperoxidase indices), reduction in the extend of muscle injury (creatine kinase level), and increased IL-10 and amino acid levels were reported (29). Athletes from a university football club (*n* = 44, multi strain probiotic, 4 weeks), showed no reduction in NK cell activity (41). In division one baseball players (*n* = 25, single strain probiotic, 12 weeks), reduced TNF-α levels were reported (44). In elite athletes from several different sports (*n* = 39, single strain probiotic, 14 weeks) increased self-rated sense of vigor and improved CD4+/CD8+ ratio (ratio of T helper cells) was demonstrated (53). In runners (*n* = 65, single strain probiotic, 15 weeks) an attenuated increase in serum CRP was found (54). University athletes and game players (*n* = 243, single strain probiotic, 20 weeks) showed a reduced cytomegalovirus and Epstein-Barr virus load (55). In soldiers of an elite combat unit (*n* = 26, single strain probiotic, 6 weeks), IL-6 but also IL-10 levels were attenuated (45). Elite distance runners (*n* = 20, single strain probiotic, 16 weeks) showed increased IFN-γ levels but no significant differences in salivary IgA/ IgA1 and serum IL-4 and IL-12 levels (51). Elite rugby union athletes (*n* = 19, multi strain probiotic, 17 weeks) showed increased salivary alpha-amylase (host defense peptide) levels (57).

No effects of probiotics on markers of the immune system were found in division one collegiate female swimmers (*n* = 17, single strain probiotic, 6 weeks) (46), elite athletes (badminton, triathlon, bicycling, athletics, karate, kayaking, and judo, *n* = 30, single strain probiotic, 14 weeks) (52), commando cadets (*n* = 47, single strain probiotic, 3 weeks) (37), runners in the heat (*n* = 8, single strain probiotic, 1 week; *n* = 10, multi strain probiotic, 4 weeks) (38, 42), ultra-marathon runners (*n* = 32, multi strain probiotic, 12 weeks) (49), endurance runners in the heat (*n* = 8, single strain probiotic, 1 week) (39) and endurance-trained men (triathletes, runners, and cyclists, *n* = 23, multi strain probiotics, 14 weeks) (56). However, a tendency for supplementation over a period longer than 12 weeks in athletes may potentially produce stronger effects on the immune system compared to shorter intervention times.

### Probiotic Supplementation in Recreational Exercise

Ten of the included studies investigated the effects of probiotics on URTI (4 studies), GI symptoms (3 studies), and/or immune system (8 studies) in recreationally active individuals. Outcomes have been evaluated after supplementation for <5 weeks (*N* = 5), 5–12 weeks (*N* = 4) and >12 weeks (*N* = 1). Two studies found significant positive effects of probiotics on URTI, one on GI symptoms and 5 on the immune system and inflammatory response (Figure 2).

#### Effects on URTI

Four of the 10 studies investigated the effects of probiotic supplementation on URTI, two of which showed significant positive effects (Table 2). In students at a university sports club (*n* = 51, single strain probiotic, 13 days), significant lower days of URTI were reported (59). Participants involved in endurance activities (*n* = 84, single strain probiotic, 16 weeks) reported a significant reduction of URTI episodes (63). No effects were reported in amateur marathon runners (*n* = 42, single strain probiotic, 4 weeks) (58) and in individuals training for a marathon (*n* = 141, single strain probiotic, 12 weeks) (62).

#### Effects on GI Symptoms

Three of 10 studies investigated the effect of probiotic supplementation on GI markers and symptoms, one of which showed significant positive effects. Recreational marathon runners (*n* = 24, multi strain probiotic, 4 weeks) reported lower incidence and severity of GI symptoms (60). No effects were reported in individuals training for a marathon (*n* = 141, single strain probiotic, 12 weeks) (62) and recreational triathletes (*n* = 30, multi strain probiotic, 12 weeks) (10).

#### Effects on the Immune System

Eight of 10 studies investigated the effect of probiotic supplementation on the immune system, 5 of which showed significant positive effects. Supplementing amateur marathon runners (*n* = 42, single strain probiotic, 4 weeks) improved airway and systemic immune system/inflammatory response in that IL-10 levels were increased, TNF-α levels were decreased, and lower neutrophil infiltration was found post-marathon (58). Amateur cyclists (*n* = 42, multi strain probiotic, 4 weeks) showed reduced oxidative stress, as plasma antioxidant levels increased (23). In recreational triathletes (*n* = 30, multi strain pro/prebiotic/antioxidant, 12 weeks) reduced endotoxin levels were reported (10). In students at a university sports club (*n* = 51, single strain probiotic, 2 weeks), CD86, a maturation marker on plasmacytoid dendritic cells (pDC), was significantly increased (59). Recreational endurance-trained individuals (*n* = 84, single strain probiotic, 16 weeks) showed higher saliva IgA concentrations (63).

No effects were seen in recreational resistance-trained men (*n* = 15, single strain probiotic, 2 weeks) (71), non-elite marathon runners (*n* = 141, single strain probiotic, 12 weeks) (61), and recreational marathon runners (*n* = 119, single strain probiotic, 12 weeks) (11).

### Probiotic Supplementation in Healthy Humans

Seven of the included studies investigated the effects of probiotics on URTI (2 studies), GI symptoms (3 studies), and/or the immune system (5 studies) in recreationally active individuals. Outcomes were evaluated after supplementation for <5 weeks (*N* = 4), 5–12 weeks (*N* = 2) and >12 weeks (*N* = 1). One study found significant positive effects of probiotics on URTI, one on GI symptoms and none on the immune system and inflammatory response (Figure 2).

#### Effects on URTI

Two of the 7 studies investigated the effect of probiotic supplementation on URTI markers and symptoms. One showed significant positive effects (Table 2). Supplementing active individuals (*n* = 465, multi strain probiotic, 5 months) led to a significant reduction of URTI episodes (69). No effects on URTI severity were reported in healthy adults (*n* = 30, single strain probiotic, 4 weeks) (64).

#### Effects on GI Symptoms

Three of the 7 studies investigated the effect of probiotic supplementation on GI markers and symptoms with one study reporting significant positive effects. In untrained subjects (*n* = 19, single strain probiotics, 4 weeks), a lower increase in intestinal fatty acid-binding protein (I-FABP), indicating improved intestinal barrier function, and thiobarbituric acid reactive substances (TBARS), indicating reduced lipid peroxidation was detected after exercise (65).

No significant effects on GI markers or symptoms were found in active individuals (*n* = 22, multi strain probiotics, 3 weeks) (68). Another study with healthy physically active individuals (*n* = 465, multi strain probiotic, 5 months), reported an insufficient number of GI illness episodes for analysis (69).

#### Effects on the Immune System

Five studies investigated the effect of probiotic supplementation on the immune system, none of which showed significant positive effects. No effects were reported in soldiers (*n* = 16, single strain probiotic, 2 weeks) (12), in healthy adults (*n* = 30, single strain probiotic, 4 weeks) (64), active students (*n* = 11, multi strain probiotic, 4 weeks) (66), active individuals (*n* = 22, multi strain probiotic, 3 weeks) (68) and healthy sedentary young males (*n* = 48, multi strain probiotic, 12 weeks) (67).

## Discussion

The aim of this systematic review was to provide a structured summary of probiotic effects in athletes, recreationally active individuals, and healthy adults on URTI, GI symptoms and the immune system. To account for the potential effects of performance level and training intensity, studies were grouped by activity/ performance level of participants, intervention duration and investigated outcome. Strain type, mode of delivery and study type were also noted as potential effectors. The main findings of our investigation were, that the identified studies differed largely in terms of selected probiotics and probiotic strains, mode of delivery, intervention duration, selected outcome variables and measurement as well as included subjects. In athletes, the type of sport performed added to the observed heterogeneity. Further, our approach revealed that studies provided inconsistent observations without significant differences between the analyzed performance levels.

The analysis of 41 studies in the field revealed that some interventions induced positive effects in terms of URTI and GI symptom reduction, but almost half of the analyzed studies did not detect improvements independent of performance levels. In addition, longer duration of application did not show stronger effects on any analyzed outcome. Considering that protection of probiotics from dissolution or disintegration in the gastric environment may improve efficiency of the treatment (72–74), we also investigated the mode of delivery as potential confounding factor. However, comparison of encapsulated probiotics to probiotic beverages etc. did not reveal differences between the different modes of delivery, as on average only 50% of studies reported positive outcomes. With respect to the combined use of different genera and application of single vs. multi strain probiotics, an overall tendency toward a higher number of studies reporting beneficial effects with multi strain applications was seen. While this could simply be explained by the general assumption that the combination of a diverse selection of microorganisms with different properties would likely induce positive effects in a larger number of different individuals, none of the identified studies directly compared the use of single and multi strain probiotics. Thus, future RCTs should investigate if multi strain probiotics induce significantly greater effects on URTI, GI problems or the immune system in sport and exercise. In addition, the fact that probiotic benefits are strain-specific and some strains are more likely to improve gut or immune health in athletes than others (9), adds largely to the limited comparability between studies and complicates the interpretation of studies administering multi strain probiotics.

Considerable heterogeneity between studies was detected also in terms of the outcome variables used and their methodical assessment. However, even in studies that included identical outcome variables, no distinct effect of probiotics emerged. For example, no effects on S-IgA concentrations were detected after 3 weeks (37) or 4 weeks (51) of probiotic supplementation. Since another study reported increased S-IgA concentration after 16 weeks of probiotic supplementation (63) it could be speculated that a short-term supplementation was ineffective to induce significant effects on S-IgA levels. However, a 14-week probiotic supplementation also did not result in an elevation of S-IgA levels (52).

In terms of URTI, a number of well-designed RCTs that investigated the effects of probiotics in athletes, provided evidence that supplementation may lead to fewer days and reduced severity of URTI (40, 51, 53). These reports are opposed to studies in athletes, which reported no effects on respiratory infection incidence or symptom duration (37–39, 42, 46, 49). Of note, all identified studies which assessed the effects of probiotics on URTI, used self-assessment and self-reporting by diary or unvalidated questionnaires. Self-assessment and self-reporting were also the standard for the assessment of probiotic effects on GI symptoms, potentially contributing to opposing results even in individuals at comparable performance levels. For example, no effect on GI incidence and severity was reported after 6-week probiotic supplementation in elite rugby union players (57), while supplementing runners for 4 weeks prior to a marathon race with probiotics was suggested to reduce resting GI symptoms and severity (60). In addition, the time point (resting vs. post-exercise) or individual characteristics may have affected the observed results. To this respect, it has been speculated that GI symptoms may be more pronounced in runners who are unable to maintain their race pace, which elicits negative emotions potentially affecting the GI system (75).

In general, a significant level of heterogeneity in the field resides in the selection of analyzed subjects and their respective performance and training status. While athletes may be more susceptible to GI problems than healthy moderately-trained or sedentary adults not exposed to intense training conditions, athletes might benefit considerably from the use of probiotics (76). However, our analysis provides evidence that also in athletes, the effects of probiotics on GI symptoms are unclear and the percentage of studies reporting positive effects tended to be identical or even lower compared to individuals at lower performance levels. Of note, studies that involved objective markers of gastrointestinal barrier function such as zonulin and I-FABP did not detect significant effects of probiotics. However, a 4-week probiotic supplementation in healthy individuals was reported to lead to a reduced increase in acute I-FABP and TBARS after exercise, suggesting that results may differ also depending on the time point of assessment (65).

In terms of probiotic supplementation on immune system function, some studies found significant acute effects of probiotics such as reduced acute NK cell activity post-exercise (41) as well as chronic effects including an increase in microbial diversity (36) or reduced resting TNF-α, IL-6 and IL-8 levels (29). Of note, even within study types (RCT vs. longitudinal studies) no consistent effect was observed. In addition, no type of sport or exercise with specific stressors in which probiotics could have been particularly effective was identified, even if studies on endurance-trained athletes or resistance-trained athletes were compared. Of note, out of five studies investigating the effects of probiotics on the immune system in healthy individuals, no study detected significant improvements, indicating that performance level could be a relevant classifier in this aspect.

### Limitations

Some limitations for the presented analysis may exist. Reporting and publication bias may have affected the present review since some data/ studies may have remained unreported or were not published because of unexpected/ contradictory, negative or not significant test results. Furthermore, the record search was limited to studies published in English and inclusion of data reported in other languages may have altered the results preliminary in subgroups with smaller sample sizes.

### Conclusion and Perspective

While athletes may be more susceptible to GI problems and URTI due to the immunosuppressive effects of repeated intensive exercise (76, 77), recreational exercise does not induce immunosuppressive effects in healthy adults and the beneficial effects of probiotics may thus be less obvious in this group. Since the number of available studies in healthy adults is currently rather limited, this aspect requires further investigation. Currently, due to overall inconsistent observations, the use of probiotics in sport and exercise remains inconclusive. In addition, further studies investigating the effect of probiotics on acute exercise-induced changes of immune function markers are mandatory to identify the potential effects of probiotics in physically active individuals at different performance levels. Moreover, the effects of probiotics in women need further examination, since available studies predominantly included male subjects and one study indicated sex-specific differences, reporting a significant reduction in duration and severity of respiratory illness in males, but not in females (68). In general, the heterogeneity in the documentation of GI illness and URTI incidence, duration, severity, and symptoms by self-reporting adds further uncertainty to the evaluation of potential beneficial probiotic effects. A concerted approach to definition, diagnosis, and documentation, as well as to assessed variables would help to obtain comparable results. This should include the use of validated questionnaires such as the Gastrointestinal Symptom Rating Scale (GSRS) (70) for GI problems and the Wisconsin Upper Respiratory Symptom Survey (WURSS) (78) for URTI. In addition, it might be necessary to include serological diagnosis of URTI instead of using symptomatic monitoring as recently suggested (48). Since a correlation between an increase in the number of respiratory illnesses and a decrease in s-IgA during training has been observed (79), s-IgA may be a useful marker with some potential to investigate the connection of immune system function and URTI. Since GI permeability has been shown to be increased following strenuous exercise also without apparent self-reported GI symptoms (80) serum markers of GI barrier integrity/ intestinal permeability including zonulin should be included in future studies on probiotic supplementation in exercise.

## Data Availability Statement

The original contributions presented in the study are included in the article/Supplementary Materials, further inquiries can be directed to the corresponding author.

## Author Contributions

MH and FM contributed to the conception and design of the study. MH performed the systematic literature search. MH, BS, and MT screened records and extracted data. MH, FM, and BS interpreted the data. MH and BS wrote the manuscript. All authors contributed to the drafting, revision of the manuscript, and approved the final version of the manuscript.

## Funding

FM and BS are supported by the European Commission within the Horizon 2020 framework program (grant number: 101017424).

## Conflict of Interest

The authors declare that the research was conducted in the absence of any commercial or financial relationships that could be construed as a potential conflict of interest.

## Publisher's Note

All claims expressed in this article are solely those of the authors and do not necessarily represent those of their affiliated organizations, or those of the publisher, the editors and the reviewers. Any product that may be evaluated in this article, or claim that may be made by its manufacturer, is not guaranteed or endorsed by the publisher.
