# Corrigendum: Health benefits of probiotics in sport and exercise—Non-existent or a matter of heterogeneity? A systematic review

**DOI:** 10.3389/fnut.2022.1051918

**Published:** 2022-10-17

**Authors:** Melina Heimer, Marc Teschler, Boris Schmitz, Frank C. Mooren

**Affiliations:** ^1^Department of Rehabilitation Sciences, Faculty of Health, University of Witten/Herdecke, Witten, Germany; ^2^DRV Clinic Königsfeld, Center for Medical Rehabilitation, Ennepetal, Germany

**Keywords:** exercise, gut microbiota, respiratory infection, immune system, nutrition, immunology, probiotic, gastrointestinal

In the published article, there was an error in the **Funding** statement. No information on funding was given. The correct **Funding** statement appears below.

## Funding

FM and BS are supported by the European Commission within the Horizon 2020 framework program (grant number: 101017424).

The authors apologize for this error and state that this does not change the scientific conclusions of the article in any way. The original article has been updated.

## Publisher's note

All claims expressed in this article are solely those of the authors and do not necessarily represent those of their affiliated organizations, or those of the publisher, the editors and the reviewers. Any product that may be evaluated in this article, or claim that may be made by its manufacturer, is not guaranteed or endorsed by the publisher.

