# Supplementary material for: Health Benefits of Probiotics in Sport and Exercise - Non-existent or a Matter of Heterogeneity? A Systematic Review
Source: Front Nutr. 2022 Feb 23;9:804046. doi: 10.3389/fnut.2022.804046 (PMC8906887; doi:10.3389/fnut.2022.804046)
Supplement: Supplementary file 1 [file Table_1.pdf]

Supplemental Table 1: Search terms and queries by database

A	PubMed
#1	probiotic*
#2	sport*
#3	exercise
#4	athlete
#5	“physical activity”
#6	recreational
#7	#2 or #3 or #4 or #5 or #6
#8	inflammation
#9	URTI
#10	immunology
#11	„gut microbiota“
#12	“immune system”
#13	“gastrointestinal disease”
#14	“respiratory infection”
#15	#8 or #9 or #10 or #11 or #12 or #13 or #14
#16	child*
#17	#1 and #7 and #15 not #16
Syntax: ((probiotic*)) AND (((((sport*) OR (exercise)) OR (athlete)) OR ("physical activity")) OR (recreational)) AND (((((((inflammation) OR (URTI)) OR (immunology)) OR ("gut microbiota")) OR ("immune system")) OR ("gastrointestinal disease")) OR ("respiratory infection")) NOT child*	

B	Web of Science
#1	probiotic*
#2	sport*
#3	exercise
#4	athlete
#5	“physical activity”
#6	recreational
#7	#2 or #3 or #4 or #5 or #6
#8	inflammation
#9	URTI
#10	immunology
#11	„gut microbiota“
#12	“immune system”
#13	“gastrointestinal disease”
#14	“respiratory infection”
#15	#8 or #9 or #10 or #11 or #12 or #13 or #14
#16	child*
#17	#1 and #7 and #15 not #16
Syntax: ALL=(probiotics*) AND ((((ALL=(sport*)) OR ALL=(exercise)) OR ALL=(athlete)) OR ALL=("physical activity")) OR ALL=(recreational) AND (((((((ALL=(inflammation)) AND ALL=(immunology))) OR ALL=(URTI)) OR ALL=("gut microbiota")) OR ALL=("immune system")) OR ALL=("gastrointestinal disease")) OR ALL=("respiratory infection") NOT ALL=(child*)	

C	**SPORTDiscus with Full Text**
#1	probiotic*
#2	sport*
#3	exercise
#4	athlete
#5	“physical activity”
#6	recreational
#7	#2 or #3 or #4 or #5 or #6
#8	inflammation
#9	URTI
#10	immunology
#11	„gut microbiota“
#12	“immune system”
#13	“gastrointestinal disease”
#14	“respiratory infection”
#15	#8 or #9 or #10 or #11 or #12 or #13 or #14
#16	child*
#17	#1 and #7 and #15 not #16

Syntax: probiotic* AND (sport* OR exercise OR athlete OR "physical activity" OR recreational) AND (inflammation OR URTI OR immunology OR "gut microbiota" OR “immune system” OR "gastrointestinal disease" OR “respiratory infection) NOT child*
