# Supplementary material for: Health Benefits of Probiotics in Sport and Exercise - Non-existent or a Matter of Heterogeneity? A Systematic Review
Source: Front Nutr. 2022 Feb 23;9:804046. doi: 10.3389/fnut.2022.804046 (PMC8906887; doi:10.3389/fnut.2022.804046)
Supplement: Supplementary file 2 [file Table_2.pdf]

Supplemental Table 2: Risk of bias assessment

Study	PEDro final score	Eligibility criteria specified	Random allocation	Concealed allocation	Similar groups at baseline	Blinding of therapists	Blinding of assessors	Blinding of subjects	Measure one key outcome from 85% subjects	Intention-to-treat analysis	Between group statistical analysis for at least one outcome	Variability and point measurements for at least one outcome
**Salehzadeh (2015)**	6/10	Yes	Yes	No	Yes	No	No	No	Yes	Yes	Yes	Yes
**Brennan et al. (2018)**	10/10	Yes	Yes	Yes	Yes	Yes	Yes	Yes	Yes	Yes	Yes	Yes
**Tiollier et al. (2007)**	10/10	Yes	Yes	Yes	Yes	Yes	Yes	Yes	Yes	Yes	Yes	Yes
**Huang et al. (2019)**	10/10	Yes	Yes	Yes	Yes	Yes	Yes	Yes	Yes	Yes	Yes	Yes
**Gill et al. (2016a)**	10/10	Yes	Yes	Yes	Yes	Yes	Yes	Yes	Yes	Yes	Yes	Yes
**Gill et al. (2016b)**	10/10	Yes	Yes	Yes	Yes	Yes	Yes	Yes	Yes	Yes	Yes	Yes
**Haywood et al. (2014)**	10/10	Yes	Yes	Yes	Yes	Yes	Yes	Yes	Yes	Yes	Yes	Yes
**Sashihara et al. (2013)**	10/10	Yes	Yes	Yes	Yes	Yes	Yes	Yes	Yes	Yes	Yes	Yes
**Shing et al. (2014)**	10/10	Yes	Yes	Yes	Yes	Yes	Yes	Yes	Yes	Yes	Yes	Yes
**West et al. (2011)**	10/10	Yes	Yes	Yes	Yes	Yes	Yes	Yes	Yes	Yes	Yes	Yes
**Townsend et al. (2018)**	10/10	Yes	Yes	Yes	Yes	Yes	Yes	Yes	Yes	Yes	Yes	Yes
**Gepner et al. (2017)**	10/10	Yes	Yes	Yes	Yes	Yes	Yes	Yes	Yes	Yes	Yes	Yes
**Carbuhn et al. (2018)**	10/10	Yes	Yes	Yes	Yes	Yes	Yes	Yes	Yes	Yes	Yes	Yes
**Charlesson et al. (2013)**	5/10	Yes	No	No	Yes	No	No	No	Yes	Yes	Yes	Yes
**Strasser et al. (2016)**	10/10	Yes	Yes	Yes	Yes	Yes	Yes	Yes	Yes	Yes	Yes	Yes
**Marshall et al. (2017)**	6/10	Yes	Yes	No	Yes	No	No	No	Yes	Yes	Yes	Yes
**Salarkia et al. (2013)**	6/10	Yes	Yes	No	Yes	No	No	No	Yes	Yes	Yes	Yes
**Cox et al. (2010)**	10/10	Yes	Yes	Yes	Yes	Yes	Yes	Yes	Yes	Yes	Yes	Yes
**Michalickova et al. (2017)**	10/10	Yes	Yes	Yes	Yes	Yes	Yes	Yes	Yes	Yes	Yes	Yes
**Michalickova et al. (2016)**	10/10	Yes	Yes	Yes	Yes	Yes	Yes	Yes	Yes	Yes	Yes	Yes
**O’Brien et al. (2015)**	6/10	Yes	Yes	No	Yes	No	No	No	Yes	Yes	Yes	Yes

**Gleeson et al. (2016)**	10/10	Yes	Yes	Yes	Yes	Yes	Yes	Yes	Yes	Yes	Yes	Yes
**Lamprecht et al. (2012)**	10/10	Yes	Yes	Yes	Yes	Yes	Yes	Yes	Yes	Yes	Yes	Yes
**Pumpa et al. (2019)**	10/10	Yes	Yes	Yes	Yes	Yes	Yes	Yes	Yes	Yes	Yes	Yes
**Jäger et al. (2016)**	10/10	Yes	Yes	Yes	Yes	Yes	Yes	Yes	Yes	Yes	Yes	Yes
**Vaisberg et al. (2019)**	10/10	Yes	Yes	Yes	Yes	Yes	Yes	Yes	Yes	Yes	Yes	Yes
**Komano et al. (2018)**	10/10	Yes	Yes	Yes	Yes	Yes	Yes	Yes	Yes	Yes	Yes	Yes
**Pugh et al. (2019)**	10/10	Yes	Yes	Yes	Yes	Yes	Yes	Yes	Yes	Yes	Yes	Yes
**Martarelli et al. (2011)**	6/10	Yes	Yes	No	Yes	No	No	No	Yes	Yes	Yes	Yes
**Moreira et al. (2007)**	10/10	Yes	Yes	Yes	Yes	Yes	Yes	Yes	Yes	Yes	Yes	Yes
**Kekkonen et al. (2007)***	10/10	Yes	Yes	Yes	Yes	Yes	Yes	Yes	Yes	Yes	Yes	Yes
**Välimäki et al. (2012)**	10/10	Yes	Yes	Yes	Yes	Yes	Yes	Yes	Yes	Yes	Yes	Yes
**Roberts et al. (2016)**	10/10	Yes	Yes	Yes	Yes	Yes	Yes	Yes	Yes	Yes	Yes	Yes
**Gleeson et al. (2011)**	10/10	Yes	Yes	Yes	Yes	Yes	Yes	Yes	Yes	Yes	Yes	Yes
**Hoffmann et al. (2019)**	10/10	Yes	Yes	Yes	Yes	Yes	Yes	Yes	Yes	Yes	Yes	Yes
**Meng et al. (2016)**	8/10	Yes	Yes	Yes	Yes	No	No	Yes	Yes	Yes	Yes	Yes
**Mooren et al. (2020)**	5/10	Yes	No	No	Yes	No	No	No	Yes	Yes	Yes	Yes
**Muhamad & Gleeson (2014)**	5/10	Yes	No	No	Yes	No	No	No	Yes	Yes	Yes	Yes
**Ibrahim et al. (2018)**	6/10	Yes	Yes	No	Yes	No	No	No	Yes	Yes	Yes	Yes
**West et al. (2012)**	10/10	Yes	Yes	Yes	Yes	Yes	Yes	Yes	Yes	Yes	Yes	Yes
**West et al. (2014)**	10/10	Yes	Yes	Yes	Yes	Yes	Yes	Yes	Yes	Yes	Yes	Yes

* Identical participants reported by Moreira et al. (2007).
